# A Holistic Assessment of the Risks and Benefits of the Synthesis of Horsepox Virus

**DOI:** 10.1128/mSphere.00074-18

**Published:** 2018-03-07

**Authors:** Diane DiEuliis, Gigi Kwik Gronvall

**Affiliations:** a Center for the Study of Weapons of Mass Destruction, National Defense University, U.S. Department of Defense, Washington, DC, USA; b Johns Hopkins Center for Health Security, Johns Hopkins Bloomberg School of Public Health, Baltimore, Maryland, USA

**Keywords:** bidoefense, biosecurity, horsepox, smallpox

## Abstract

The re-creation of horsepox virus, an extinct orthopoxvirus with similarity to smallpox virus, has caused concerns in the biosecurity and biodefense communities that the technical capabilities achieved could advance the re-creation of smallpox virus by nefarious actors. The work is now published. While the authors went through due biosecurity diligence at their research institution and with the proper Canadian federal authorities, now that the experiments have been published, there is an opportunity to discuss the dual use risks and benefits of the research itself, as well as those associated with publication of such research—all of which challenge current policies. Here, an analytical framework is used to assess the risks and benefits of such dual use research, and relevant components of biosecurity policy and the biodefense enterprise (including the acquisition of medical countermeasures) in the United States are discussed. The authors emphasize the need to use such risk/benefit assessments at the onset of research and throughout its development, followed by an assessment for its responsible communication.

## Abstract (precis)

The re-creation of horsepox virus, an extinct orthopoxvirus with similarity to smallpox virus, has caused concerns in the biosecurity and biodefense communities that the technical capabilities achieved could advance the re-creation of smallpox virus by nefarious actors. The work is now published.

## COMMENTARY

There has been considerable consternation about the chemical synthesis and “rebooting” of horsepox virus, an orthopoxvirus, now published in *PloS One* (1). Horsepox is not a significant disease for humans, but there is concern that publication of these experiments could lower barriers toward the synthesis and booting up of another orthopoxvirus, variola (smallpox) virus, which was a significant scourge in history. The current controversy about horsepox virus synthesis is an opportunity to examine (i) the challenges of forecasting risks and benefits from a particular scientific discovery or technology, (ii) how dual use risks in the scientific enterprise should be handled, and (iii) the complicated array of considerations that go into stockpiling medical countermeasures, which it is hoped will never be needed. This paper provides a dispassionate assessment of the biosecurity risks, extending earlier analyses of the biosecurity implications of horsepox virus synthesis (2), and addresses some of the primary concerns stemming from publication of the research.

## DUAL USE RISKS AND BENEFITS OF THE SCIENCE SHOULD BE ASSESSED AT THEIR ONSET

Experiments readily identified as dual use research of concern should trigger additional review of the risks and benefits at the onset of research. Identification of something as dual use should not trigger immediate denial of the project, but rather a careful consideration of whether risks outweigh benefits, and further, how risks can be best mitigated (minimized) to maximize scientific benefits. This process itself should increase security, but may also lead to valuable or innovative scientific methodology in laboratory experimentation. Dual use risks cannot always be avoided, which was described at length in the Fink report (3), but in many cases they may be mitigated through purposeful, front-end analysis.

With regard to the benefits of such research, Noyce et al. (1) hypothesized that the original smallpox vaccine devised by Jenner was based on a horsepox virus strain, and so they resolved to test horsepox virus as a potentially superior vaccine candidate for smallpox (see the discussion of medical countermeasures below). They hypothesized that the slower-growing horsepox virus would cause fewer harmful side effects than the current vaccine (which may cause cardiac events in some recipients [4]) and may not require 2 doses (which another smallpox vaccine, MVA [modified vaccinia Ankara], requires). There may also be also other potential benefits of synthesizing horsepox virus, including its use as a novel vector for development of vaccines against diseases (poxviruses have been used as a vaccine vector), use as a vector for oncolytic viruses to treat cancer, such as those pursued in the Evans laboratory, use as a vaccine against other poxviruses, including monkeypox, which is still in circulation and which causes disease in humans (5), and also the potential development as a novel vector for development for gene therapy and/or gene delivery. It should also be noted that the rapid synthetic creation of a large virus vaccine could be needed in the event of a zoonotic outbreak, in order to develop all the tools needed for an infectious disease response.

The future therapeutic success of these explorations cannot be fully predicted or where they may lead in terms of benefits, but the scientific enterprise has traditionally erred on the side of openness to a wide range of innovations. Any *a priori* determination that these proposals would not have scientific or therapeutic value (and should not be pursued) must also consider the potential negative general effects of making that determination.

## THE IMPERIALE REPORT FRAMEWORK

To assess the potential biosecurity risks of horsepox virus synthesis, the authors note that the recently published interim National Academies Imperiale report (6) offers a timely framework to systematically evaluate this technological capability described in the *PloS One* article. As stated in the report, factors for assessing the capability for malicious use include:

- The use of the technology itself
- Its potential use as a weapon
- The attributes of actors who could command such a capability

These must be weighed against the following factors to assess the capability for mitigation:

- Deterrence and prevention
- The ability to recognize an attack
- The ability to achieve attribution
- Consequence management

Several points should be highlighted. Regarding technology, Noyce et al. surmounted high technical barriers to horsepox virus synthesis. The first was the assembly and booting up of such a large-size viral genome, and the second was the recovery of infectious virus from cells; the technical demands would eliminate several types of nefarious actors were this to be done for harm (2). The authors note that since the 2010 synthesis and booting up of a bacterium, *Mycoplasma mycoides* (7), which is much larger than horsepox virus, the ability to synthesize and boot up any virus was not in doubt. As noted in our prior analysis (2), Noyce et al. used specialized terminal-end vaccinia virus DNA constructs to assist in the construction of horsepox virus segments. They added “helper” virus to the cells to recover the infectious virus, methods they had previously used successfully for vaccinia virus (8), so that published component was not novel and previously described. These horsepox experiments likely worked well due to the similarity between horsepox and vaccinia orthopoxviruses; while horsepox virus shares high sequence similarity with smallpox virus, it shares few genes in common with smallpox virus. Sequence similarity can indicate the two viruses emerged from a common evolutionary ancestor, but the horsepox and smallpox viruses appear to be phylogenetically and genomically distinct (9). Given that host specificity in poxviruses is determined by a variety of genes—not just one—a single synthesis approach for one poxvirus may not work for another phylogenetically distinct poxvirus. This suggests that there is no guarantee that the specialized tools used to synthesize horsepox virus would equate to a successful “recipe” for functional, infectious, smallpox virus, although it is likely that an experienced virologist could recreate smallpox virus through purposeful effort and continuous trial and error. Thus, tacit knowledge, specifically designed tools, specialized expertise, and a dedicated time investment for research and development (R&D) would be required for recreating smallpox virus using these methods. If an actor were able to overcome these barriers—they would still need to create a way to infect humans with the laboratory-created material, which in itself is not trivial, and potentially only surmountable by sophisticated actors.

The other component of the framework assessment involves mitigation. In terms of prevention, customer orders of synthetic DNA fragments resembling smallpox virus should be flagged by DNA providers through sequence screening, and those orders would be further scrutinized. The customer and his affiliated organization should also be screened—it is presumed that both of these screening steps occurred for the work performed by Noyce et al. However, the limits of such screening have been recognized by the authors (10) and others (11, 12), and it is therefore not guaranteed that screening provides a full prevention measure. The sequence similarity issue arises here as well; while a BLAST search clearly distinguishes horsepox virus from smallpox virus, it is not clear how tailored screening algorithms used by companies that screen their orders distinguish the sequences. Horsepox virus is not on any select agent lists or regulated pathogen lists, which form the basis of sequence screening (13).

In summary, the ability to chemically synthesize any virus poses biosecurity risks, many of which have been analyzed against current United States policies (2). There are no 100% solutions to this dilemma, but there are several partial solutions (such as those described in the above referenced paper on screening), and more need to be developed. If anything, this controversy has raised awareness that more “partial solutions” need to be implemented to raise prevention barriers for nefarious actors who would wish to use these technologies in creating a virus.

If a re-created smallpox were somehow delivered to a human population and proved infectious, it would be rapidly detected: in fact, as an eradicated disease, its reappearance would likely provoke an instantaneously triggered biodefense response. There are enough doses of smallpox vaccine in the Strategic National Stockpile (SNS) to vaccinate most of the U.S. population, if necessary, and multiple other nations also have stockpiles—a powerful deterrent to the use of smallpox by adversaries. This mitigation needs to be weighed against the risks noted above.

## IS A NEW SMALLPOX VACCINE NEEDED?

The decision of whether the U.S. Government (USG) should invest in a medical countermeasure (MCM) is multifactorial, reflecting the changing bioweapons and bioterrorism threat, the long development time for medical countermeasures, advances in biotechnology that may make a future vaccine or drug a more appropriate investment, the availability of a current technology that can offer protection, availability of funds, the concept of operations for the MCM use in an emergency, and the inevitable expiration of already created MCMs in the current stockpile. Dynamic changes in all of these variables will affect whether the USG will invest in a particular MCM. The Public Health Emergency Medical Countermeasures Enterprise (PHEMCE) is the United States interagency deliberative group responsible for making decisions on the development of MCMs for the United States Strategic National Stockpile (14), and the Biomedical Advanced Research and Development Authority (BARDA) is the agency responsible for making countermeasure investments.

It has proven difficult to interest companies in developing MCMs for biodefense when the USG is the only buyer. Thus, the USG continues to search for better incentives for gaining industry interest in developing MCMs, such as hosting monthly “TechWatch” calls to encourage companies to present any promising technology advancements and emerging opportunities in the MCM space. The new expedited review voucher program created by the 21st Century Cures Act is also meant to incentivize pharmaceutical and biotechnology companies to produce MCMs for biosecurity threats (15).

MCM development is not a static process, particularly in the context of emerging technology and evolving threats. Pathogens evolve, there is potential for unexpected zoonoses, creation of altered or novel pathogens is now possible, and human hosts have unique immunological characteristics, all of which necessitate some flexibility in the preparedness approach. The PHEMCE has recognized this, and it is consistent with a critical strategic pivot made by the PHEMCE since its initial creation—that is, a focus away from threat-based countermeasure development to one which is capabilities based (16). For example, a “universal” influenza vaccine which could combat numerous potentially pandemic strains would be much more advantageous, efficient, and less expensive than developing many individual influenza vaccines; essentially, this single capability could counter a myriad of individual threats. Similarly, broad-based antibacterial drugs are desperately needed, and national-level strategies (17) have articulated the need for innovative approaches in their creation. In addition, all items in the stockpile will eventually expire and will need to be replaced—and they may not necessarily be replaced with the same technologies of 10 or more years prior. The pace of biotechnology advances rapidly, opening the possibility of novel approaches. BARDA balances many priorities when considering how to invest in medical countermeasures, but it is a mischaracterization of their mission to assume that once a vaccine has been purchased, they will not invest in additional or novel technologies in the future. An example is the investment in monoclonal antibody approaches; these were disappointing at their inception, but now show much greater promise in light of advancing technology (18).

In 2016, the PHEMCE approved the Smallpox Vaccine Response Strategy (19), which provides information on decisions regarding the effective use of smallpox vaccines in the SNS after the detection of smallpox disease. Based on this, the PHEMCE will maintain sufficient quantities of smallpox vaccines in the SNS to provide a response capability to vaccinate every American during a smallpox emergency, if appropriate, including use of a vaccine for at-risk populations, if necessary. BARDA is pursuing the potential use of a safer vaccine in such populations, and the PHEMCE Strategic Implementation Plan calls for a systematic review of the literature to determine if pursuit of antivirals should augment existing smallpox MCMs in the stockpile (20).

The PHEMCE will come to its own scientific and technical determination of whether a new smallpox vaccine based on horsepox virus is necessary or feasible in view of these efforts, its other MCM priorities, and its limited budget. However, it should be considered that other components of the stockpile are aimed at addressing adverse events associated with the present smallpox vaccine—not only the new vaccine being pursued for at-risk populations but also the stockpiling of vaccinia immune globulin intravenous (VIGIV) and the funding of associated animal models to test the efficacy of VIGIV or other therapeutics over time to address adverse events in those vaccinated (21). The maintenance of a vaccine for the stockpile that has side effects and is not safe to use in at-risk populations necessitates other costs associated with mitigation of those features.

Finally, maintenance of any smallpox MCM in the SNS requires smallpox-specific, validated diagnostic tools, the ability to test the efficacy of aging or newly manufactured reagents over time against smallpox, and the creation of validated animal models for the same and for the testing of varied formulations; these requirements continue to support the ongoing maintenance of original stocks of smallpox virus at the CDC. The ability to rapidly synthesize vaccine viruses in the laboratory would be of utility during an infectious outbreak. Experiments such as those performed with horsepox virus could yield tools for use in this context, and vaccine development research in general adds to the knowledge base that provides benefit to biodefense.

## SHOULD THE HORSEPOX EXPERIMENTS HAVE BEEN PUBLISHED?

Several years ago, an international “dual use research of concern” (DURC) policy discussion was triggered over “gain-of-function” experiments when two research investigators submitted articles that identified specific genetic substitutions and other methodologies that potentially enhanced the functions of the highly pathogenic H5N1 influenza virus strain (22). This revealed a two-fold problem for biosecurity: (i) the research was ready to be published before government officials were fully aware of the outcomes of the experiments, and (ii) publication of the experiments contained dual use information.

In terms of the first problem, the solution was to emphasize transparency and awareness of potential dual use research of concern at all levels of the research infrastructure, and most particularly at the very inception of DURC research, although this has been recognized as challenging (23). This included policies to address the role of the Federal Government (24) in reviewing proposed research, as well as the role and responsibility of the individual research institution (25). These approaches are focused on select agents and identified experiments of concern, and more recently a specific policy was released to assist in risk/benefit assessments for funding decisions for gain-of-function research in pathogenic influenza virus strains (26). Although the horsepox virus synthesis did not occur in the United States, a number of front end research reviews were done, including discussions with the Alberta Institutional Biosafety Committee (IBC), Animal Care and Use, and consultation with Canadian Government officials in the Ministry of Health, but given that horsepox virus is not a regulated pathogen, it would not necessarily be captured by United States DURC policies if the research had been done in the United States. The experiments do offer an opportunity to emphasize DURC transparency and awareness so that risk/benefit assessments can be made with input from a variety of experts in academia, MCM development, and biodefense/biosecurity at their onset.

The controversy surrounding the H5N1 experiments also highlighted a recurring problem in the life sciences related to the sharing of dual use information, which to date, remains largely unresolved. Deliberations of the National Science Advisory Board for Biosecurity (NSABB), at the time the H5N1 experiments were submitted for publication, revisited the tension between national security and openness in the life sciences originally debated by the National Academies in the Corson report (27). At that time, the USG maintained high security concerns about the leaking of U.S. technologies to foreign entities. As a result, the U.S. Department of Defense and other funding agencies incorporated routine restrictions on publication or other dissemination of research results as a condition of contractual funding. Deemed “troublesome clauses” by those in the research community, concerns were expressed that these restrictions hampered discoveries and advancement of the very research that the USG sought to protect. The result was National Security Decision Directive 189 (NSDD-189) (28), which states that “to the maximum extent possible, the products of fundamental research remain unrestricted” and further, “No restrictions may be placed upon the conduct or reporting of federally funded fundamental research that has not received national security classification, except as provided in applicable U.S. Statutes.” NSDD-189, originally signed by President Reagan, was renewed in support by President Bush shortly after the events of 9/11 and remains in effect.

Given the binary choice between openness versus classification, nearly all research is done without restriction. In fact, classification of biological research can only occur under certain proscribed conditions, so as not to jeopardize compliance with the Biological Weapons Convention (BWC); research in the context of biodefense occurs only in select research institutions, such as the National Biodefense Analysis and Countermeasures Center (NBACC), and only when there is actionable intelligence of a threat (29).

The NSABB struggled with this challenge. They recognized that the gain-of-function influenza virus research had been cited as being very useful to flu surveillance—thus they tried to devise a communication mechanism for sharing information about which mutations could increase transmissibility for those with a “need to know” in the public health community. However, such constructs based on an “experts only” mechanism of communication proved to be unviable. That said, the NSABB recognized that DURC information still represents risks, and mitigation steps could be taken to minimize the risk that a nefarious actor would utilize it for harm; this needs to be balanced against the need for the research to be replicated by others in order to substantiate its validity. The DURC Policy’s “Companion Guide” (30) offers guidance and a decision tree for the publication of DURC research information intended to mitigate such risks, and the authors deem it could be useful for both research investigators, and journal biosecurity review committees. The National Academies of Science recently assessed these dual use dilemmas and offer additional suggestions for how dual use research results can be disseminated (31).

Once again, the opportunity costs of not publishing must be taken into account. In the United States, if research information is not publicly shared through open publication, it becomes subject to United States Export Control, and so a license would be required for sharing or even verbal discussions of such information with individuals outside the United States. This can generate a perception of lack of compliance with the BWC. (In the aforementioned example case of the H5N1 experiments, U.S.-imposed export controls would have blocked information from international partners in the public health community and among members of the Pandemic Influenza Partnership through the World Health Organization. For a full summary of NSABB deliberations on this wide-ranging topic, see information in reference 32).

With regard to the horsepox experiments, as we have noted above, the methodology described is not a “recipe” *per se* for smallpox, although it provides general guidance for experienced actors to attempt smallpox virus construction. Given much of this information already exists in the literature, the publication of the smallpox experiments may only provide additional instructive capability to experienced actors. Within scientific research publications, methods sections are generally high level and describe a path followed, versus a step-by-step recipe. While this may lead to challenges in the reproducibility of scientific results, it selects for a certain type of actor who might misuse this information.

## CONCLUSIONS

This paper is meant to provide an assessment of the biosecurity risks incurred by the specific horsepox research, and its publication. The authors previously analyzed the biosecurity implications of the horsepox virus synthesis prior to its publication and determined that a high degree of technical ability and tacit knowledge were required, which eliminates many types of nefarious actors who might misuse this information. Now that the work has been published, the authors examined the research according to the Imperiale report framework, which aims to provide a systematic way to evaluate biosecurity risks. We again found that while dual use information would benefit highly experienced actors who are intent on misuse, the recreation of smallpox virus may require additional research and development steps than have been described in this publication: smallpox virus is less similar to horsepox virus than horsepox virus is to vaccinia virus, the tools to recreate horsepox virus were originally developed for vaccinia virus, and they might require additional troubleshooting for re-creation of smallpox virus. An experienced actor would surmount these issues, but they would take additional time and funds.

Nonetheless, there are issues raised by the horsepox paper which will require additional work and further consideration by policy experts.

### (i) The decision of what to do with a technology or research area that is dual use cannot be black or white.

The potential benefits of the scientific enterprise can be immediate or far-reaching. There are steps that might be taken to diminish the potential risks and opportunities to communicate why it might be important to go ahead in spite of those risks. While it is hard to have a full accounting of the benefits, deciding to not engage in a particular research area could also pose risks. The idea of a risk/benefit analysis for every experiment is appealing, but a comprehensive approach to each dual use issue would be difficult to accomplish in practice. Nonetheless, before one calls for a moratorium on a particular area of research or that it should be under an international control mechanism, there should be an examination for untoward effects.

### (ii) MCMs cannot be a check-the-box procedure for the USG.

If use of smallpox remains a biosecurity risk into the future, new smallpox medical countermeasures are likely to be developed and purchased. We advocate a combination of scientific risk/benefit assessment, use of policies to mitigate risk, and a dynamic and open approach to MCM development that maximizes preparedness as threats evolve. As the stockpile needs to be refreshed as medical countermeasures expire, as technology opportunities improve, and as the threat space is in flux, the USG is not going to be “done” with MCMs for a particular threat if the threat is ongoing.

### (iii) The synthesis of and booting up of a pathogen should serve as strategic warning that current biosecurity controls and preparedness are insufficient.

The biosecurity risks posed by the specific publication of the horsepox virus experiments are not appreciably heightened for most types of actors. In addition, there are potential benefits to the work that while challenging to evaluate, are compelling enough so that any calls for bans or changes to the regulatory framework should be carefully evaluated before implementation. Nonetheless, there are biosecurity risks that should not be ignored, but have not been the focus of controversy in this case. The chain of events that led from the publication of the smallpox virus genetic sequence in 1994, synthesis and booting up of multiple viruses, including polio virus in 2002, a bacteriophage in 2003, 1918 influenza virus in 2005, and the synthesis of a bacterial cell in 2010 were all legitimate research efforts and have led to demonstrable benefits. But now, the inevitable consequence for biosecurity is that the synthesis of any virus is within reach given a variable amount of R&D. As United States biosecurity preparedness is largely based on access control to a specific list of regulated pathogens, this provides a workaround for nefarious actors. Future biosecurity planning should take this into account.
