# Erratum for DiEuliis and Gronvall, “A Holistic Assessment of the Risks and Benefits of the Synthesis of Horsepox Virus”

**DOI:** 10.1128/mSphere.00160-18

**Published:** 2018-04-11

**Authors:** Diane DiEuliis, Gigi Kwik Gronvall

**Affiliations:** a Center for the Study of Weapons of Mass Destruction, National Defense University, U.S. Department of Defense, Washington, DC, USA; b Johns Hopkins Center for Health Security, Johns Hopkins Bloomberg School of Public Health, Baltimore, Maryland, USA

## ERRATUM

Volume 3, no. 2, e00074-18, 2018, https://doi.org/10.1128/mSphere.00074-18. Page 6 of the PDF, third full paragraph, line 4: “smallpox” should be “horsepox.” The corrected sentence should read “Given much of this information already exists in the literature, the publication of the horsepox experiments may only provide additional instructive capability to experienced actors.”

